# Morphological Characterization and Assessment of Genetic Variability of *Tylenchulus semipenetrans* Populations from Southern Iran

**DOI:** 10.2478/jofnem-2024-0047

**Published:** 2024-12-15

**Authors:** Mohammad Rumiani, Miloslav Zouhar, Akbar Karegar, Habiballah Hamzehzarghani, Ahmad Tahmasebi, Milad Rashidifard

**Affiliations:** Department of Plant Protection, School of Agriculture, Shiraz University, Shiraz, Iran.; Department of Plant Protection, Faculty of Agrobiology, Food and Natural Resources, Czech University of Life Sciences, Prague, Czech Republic.; Institute of Biotechnology, School of Agriculture, Shiraz University, Shiraz, Iran.; Department of Zoology and Entomology, University of the Free State, PO Box 339, Bloemfontein 9300, South Africa.

**Keywords:** Citrus nematodes, COI, D2-D3 of 28S rDNA, Haplotype, ITS rDNA, Morphometric, Sequencing

## Abstract

Molecular data should be combined with morphological data to enhance the reliability of phylogenetic and diagnostic studies on nematodes. In this study, the citrus nematode *Tylenchulus semipenetrans* collected from citrus orchards in different localities in Fars province, southern Iran, was characterized using the partial sequencing of ITS rDNA, D2-D3 of 28S rDNA and COI mtDNA genes. We also morphometrically characterized the second-stage juveniles (J2) and male specimens. The results showed that *T. semipenetrans* is a genetically homogeneous species, and only minor nucleotide differences were detected among the populations. Phylogenetic studies demonstrated that most Iranian populations were grouped together, and there were no differences among the populations. However, sequence alignment of ITS, D2-D3 of 28S rDNA and COI mtDNA revealed 17, 24, and 16 single nucleotide variations (SNVs) and 11, 12, and 11 single-nucleotide polymorphisms (SNPs), respectively. The results of the morphometric analysis showed slight morphometric differences among and within the populations of *T. semipenetrans*. The morphometric differences among citrus nematode populations and the haplotype topology of the populations did not correlate with their geographical origin and host type. The constructed phylogenetic trees showed a close relationship between *Tylenchulus* and *Trophotylenchulus*. In addition, the phylogenetic relationships showed that *T. musicola* is the closest taxon to *T. semipenetrans.* The results of this study provide a better understanding of the diversity of *T. semipenetrans* populations and may shed light on the genetic variation of citrus nematode.

Application of management strategies and development of cultivars resistant to plant parasitic nematodes (PPN) requires insight into taxonomic criteria and proper molecular characterization. *Tylenchulus* species are distinguished by minor morphological and morphometric characteristics (Inserra et al., 1988; Tanha Maafi et al., 2012). However, in many cases, diversity among populations is not sufficiently discernible to be evaluated using conventional taxonomic approaches, especially in cases where morphological characteristics may lead to ambiguous interpretations. Furthermore, identification based on morphology and morphometrics is time-consuming and requires considerable expertise (Szalanski et al., 1997; Al-Banna et al., 2004; Subbotin et al., 2013). Nevertheless, taxonomic identification based on the analysis of differences in morphological characteristics also needs to be supported by molecular identification. Understanding phylogeny mostly depends on using appropriate genes in the nuclear or mitochondrial genome (Cherry et al., 1997; Power et al., 1997; Bae et al., 2008). Several regions of ribosomal DNA (rDNA), including 18S, 5.8S, and 28S genes and internal transcribed spacers (ITS), have been used to study genetic variation among and/or within nematode species (De Luca et al., 2004; He et al., 2005; Adam et al., 2007; Palomares-Rius et al., 2008; Archidona-Yuste et al., 2016; Etongwe et al., 2020; Karani et al., 2020; Subbotin et al., 2020; Clavero-Camacho et al., 2021). These molecular markers are widely used for interspecies DNA diagnostics because of their low level of intraspecific polymorphism and the availability of universal primers that can be used for most nematode species (Powers et al., 1997; Blouin, 2002; Jiang et al., 2005; Mizukubo et al., 2007; Shao et al., 2020). Mitochondrial DNA (mtDNA) has also shown to be a conserved DNA region for genetic diversity assessment and taxonomic studies of different PPNs because of its simple and stable structure and low molecular weight (Armstrong et al., 2000; Sun et al., 2005; Jeyaprakash et al., 2006; Tu et al., 2007; Tigano et al., 2010; Duan, 2013; Etongwe et al., 2020; Shao et al., 2020; Subbotin et al., 2020; Clavero-Camacho et al., 2021).

The occurrence of *Tylenchulus semipenetrans* Cobb, 1913 was first reported in California (Cobb, 1914) and was then increasingly found in different geographical regions in various citrus species, olives, persimmons, grapes, and pomegranates around the world (Inserra et al., 1980; Duncan, 2005; Rashidifard et al., 2015a). The citrus nematode has also been a matter of grave concern in Iran, especially in the Fars province, where it has been spreading rapidly, causing a gradual reduction in trees’ vigor, leading to a slow decline in heavily-infested orchards (Duncan, 2005; Ghaderi & Mokaram Hesar, 2018). According to a survey in the Fars province, more than 70% of citrus orchards are infested with this nematode (Rumiani et al., unpubl. data). Genus-specific primers based on the D2-D3 segment of 28S (Tanha Maafi et al., 2012) and species-specific primers based on the ITS region (Liu et al., 2011; Tanha Maafi et al., 2012) were designed to identify the citrus nematode.

The loop-mediated isothermal amplification (LAMP) technique has also been used for faster detection of this species using DNA extracted from soil (Lin et al., 2016; Song et al., 2017). A few studies have been conducted to investigate the genetic diversity of the citrus nematode within the species based on the PCR-RFLP method using the ITS and D2-D3 regions of the 28S genes (Wang et al., 2004; Park et al., 2009; Tanha Maafi et al., 2012).

A limited number of rDNA gene sequences of the citrus nematode have been deposited at the National Center for Biotechnology Information (NCBI). However, there is no mitochondrial gene sequence of this nematode in the database. In addition, little information is available on the genetic diversity of this nematode and its morphometric characteristics. Therefore, this study aims to morphometrically characterize citrus nematode populations collected from different localities in Fars province, southern Iran. Moreover, their genetic variability was to be investigated using the ITS-rDNA, the D2-D3 segments of the 28S rDNA, and mitochondrial cytochrome oxidase subunit 1 (COI) sequences. The phylogenetic relationships between the Iranian populations and other closely related populations and other representatives of the family Tylenchulidae in GenBank were also studied.

## Materials and Methods

### Sample collection and nematode extraction

**Figure 1:**
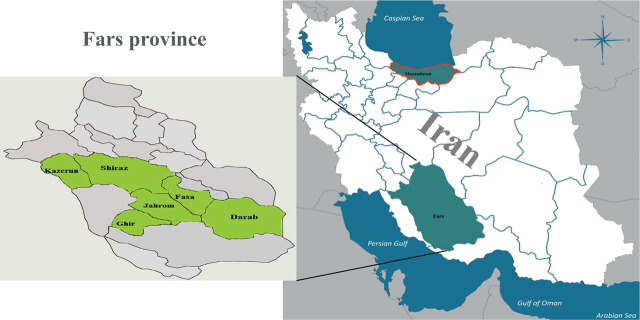
Geographical location of the sampled areas. The majority of the samples were collected in the citrus-growing regions (known as the citrus belt) of Fars province, southern Iran (shown in green: Kazerun, Shiraz, Ghir, Jahorm, Fasa and Darab). Two samples were also collected from Mazandaran province in northern Iran (shown with red border).

Nearly 60 citrus nematode-infested orchards from different citrus growing areas with different soil properties in Fars province, Iran were randomly sampled from August to October 2018 and 2019. The citrus orchards in Fars province are mainly located in six regions known collectively as the citrus belt (Fig. 1). The GPS coordinates for each sample were recorded using a GPS device (Garmin ETrex 32x) and are shown in Table 1. The majority of host trees for citrus nematode populations in the present study were orange trees (*Citrus sinensis* L.), sour orange (*C. aurantium* L.), sweet lemon (*C. limetta* R.), mandarin orange (*C. reticulata* L.) and bitter orange (*C. bigaradia* L.). Citrus trees (*Citrus* spp.) in Fars province are usually grafted onto sour orange (Ebadi et al., 2014). Indian bael (*Aegle marmelos*) is also one of the rootstocks for citrus trees in the province. In addition, some citrus trees, usually sour orange, are grown on their roots. The samples were taken from the top layer of soil, up to 30 cm below the tree canopies. To minimize the influence of the dried topsoil, the surface soil (1–3 cm) was discarded to ensure that no roots of herbaceous plants were included.

**Table 1:** The sampling sites of *Tylenchulus semipenetrans* the corresponding GenBank accession numbers for ITS, D2-D3 expansion segments of 28S and *COI* mtDNA sequences obtained in this study.

**Soil sample codes**	**GPS location**		**Locality**	**Host (*Citrus* spp.)**	**rDNA genes**		**Mt DNA COI**
	
	**latitude**	**longitude**			**D2–D3**	**ITS**	
25	28.6694	53.60647	Qotb Abad, Jahrom	*C. limetta*	OP723626	OP722727	OP739535
32	28.52135	53.67221	Jahrom	*C. sinensis*	OP723604	OP722708	OP739514
42	29.57487	51.73012	Ahmad Abad, Kazerun	*C. sinensis*	OP723627	OP722728	OP739536
112	28.95546	53.60143	Phase-e5, Fasa	*C. sinensis*	OP723629	OP722729	OP739537
411	29.62299	51.58779	Hasan Abad, Kazerun	*C. sinensis*	OP723616	OP722718	OP739524
678	28.99909	53.12713	Aliabad, Khafr	*C. aurantium*	OP723630	OP722730	-
682	28.98881	53.15637	Balashahr, Khafr	*C. sinensis*	-	-	-
698	28.51967	53.60683	Heydarabad, Jahrom	*C. limetta*	OP723597	-	OP739507
707	28.53547	53.65353	Najib Abad, Jahrom	*C. aurantium*	OP723610	OP722704	OP739510
710	28.5391	53.53022	Maghsudabad, Jahrom	*C. limetta*	OP723606	OP722696	OP739515
712	28.5397	53.53049	Maghsudabad, Jahrom	*C. aurantium*	OP723631	OP722731	OP739538
716	28.47551	53.49098	Mill, Jahrom	*C. aurantium*	OP723607	OP722710	OP739516
717	28.66863	53.60608	Qotb Abad, Jahrom	*C. limetta*	OP723591	OP722698	OP739501
720	28.66878	53.6059	Yousofabad, Jahrom	*C. limetta*	OP723592	OP722699	OP739502
733	28.93138	53.60712	Kazemabad, Fasa	*C. sinensis*	OP723601	OP722705	OP739511
735	28.97311	53.63675	Banyan, Fasa	*C. sinensis*	OP723613	OP722715	OP739521
737	28.95768	53.5981	Phase-e5, Fasa	*C. sinensis*	OP723614	OP722716	OP739522
743	29.03657	53.64304	Akbarabad, Fasa	*C. bigaradia*	OP723615	OP722717	OP739523
746	28.668855	54.665161	Bagh-e Morakabat, Darab	*C. sinensis*	OP723628	OP722722	OP739529
749	28.72201	54.57227	Naghsh Shapour, Darab	*C. sinensis*	OP723632	OP722732	OP739539
755	28.67927	54.65618	Jannat Shahr, Darab	*C. bigaradia*	OP723639	OP722738	OP739546
759	28.64105	54.64284	Deh Kheyr Payin, Darab	*C. sinensis*	OP723633	OP722733	OP739540
763	28.75331	54.44562	Sharak-e Sarollah, Darab	*C. sinensis*	OP723618	OP722719	OP739526
765	28.95749	53.60026	Hasan Abad, Darab	*C. limetta*	OP723634	-	OP739541
771	28.786689	54.339938	Fasarood, Darab	*C. aurantium*	OP723593	OP722700	OP739503
772	28.687654	54.647131	Zein Abad Sangi, Darab	*C. sinensis*	OP723608	OP722712	OP739517
773	28.76994	54.22515	Eij, Darab	*C. sinensis*	OP723602	OP722706	OP739512
777	28.445396	53.042906	Gandoman, Karzin	*C. reticulata*	OP723594	OP722701	OP739504
780	28.442301	53.143399	Emam Shahr, Ghir	*C. limetta*	OP723596	OP722702	OP739506
785	28.34352	53.25282	Tang-e Ruein, Ghir	*C. aurantium*	OP723636	OP722735	OP739543
789	28.452529	53.127036	Deh Beh, Ghir	*C. aurantium*	OP723609	OP722711	OP739518
793	28.285395	53.074062	Mand, Karzin	*C. bigaradia*	OP723619	OP722720	OP739527
795	28.328269	53.038029	Eslam Abad, Karzin	*C. aurantium*	OP723620	OP722721	OP739528
801	29.56717	51.75703	Ahmadabad, Kazerun	*C. aurantium*	OP723611	OP722713	OP739519
802	29.56924	51.75969	Ahmadabad, Kazerun	*C. aurantium*	OP723637	OP722736	OP739544
812	29.79435	51.57338	Ganjeii, Kazerun	*C. sinensis*	OP723640	OP722739	OP739547
818	29.7594	51.55155	Sheykhi, Kazerun	*C. sinensis*	OP723642	OP722741	OP739549
821	29.72973	51.53522	Anarestan, Kazerun	*C. sinensis*	OP723612	OP722714	OP739520
882	29.56109	51.77738	Zavali, Kazerun	*C. aurantium*	OP723638	OP722737	OP739545
908	29.00358	53.11102	Karadeh, Khafr	*C. aurantium*	OP723603	OP722707	OP739513
921	28.92434	53.33666	Khavaran, Khafr	*C. sinensis*	OP723641	OP722740	OP739548
740-2	28.89014	53.68479	Dastjeh, Fasa	*C. sinensis*	OP723617	-	OP739525
Ami	28.96260	54.04458	Darab	*C. aurantium*	OP723621	OP722723	OP739530
ARE	29.61910	52.57446	Sardaran, Shiraz	*C. limetta*	OP723635	OP722734	OP739542
Beh	28.47110	53.03187	Karzin	*C. aurantium*	OP723622	OP722724	OP739531
Behz-Greenhouse	29.63544	52.52485	Eram Garden, Shiraz	*C. aurantium*	OP723623	OP722725	OP739532
Sh1	36.613791	53.258824	Behshahr1, Mazandaran	*C. sinensis*	OP723624	-	OP739533
Sh2	36.625476	52.931576	Behshahr2, Mazandaran	*C. sinensis*	OP723625	OP722726	OP739534

Soil samples (approximately 1 kg each) were placed in plastic containers, labeled, and taken to the nematology laboratory at the School of Agriculture, Shiraz University. The collected samples were thoroughly mixed by hand and prepared by sieving (1.25 μm openings). Then the males and second-stage juveniles (J2) of the citrus nematode in 200 cm^3^ subsamples were extracted for 48 hours using the tray method (Whitehead & Hemming, 1965). Because of the long period between nematode extraction and molecular analysis (DNA extraction), the collected soil samples were inoculated onto sour orange rootstocks under greenhouse conditions. DESS solution (0.25 M disodium EDTA at pH 8.0, 20% dimethyl sulfoxide [DMSO], and saturated NaCl) was used to preserve the individual nematodes until molecular analysis was performed at the Nematology Laboratory of the Czech University of Life Sciences Prague (Yoder et al., 2006; Perry et al., 2020).

### Morphological studies

**Figure 2:**
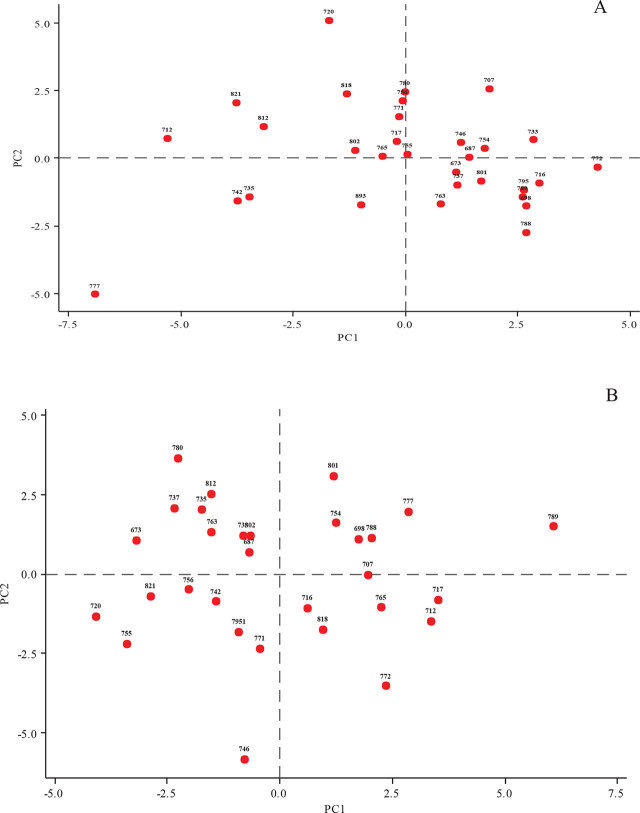
Principal Component Analysis (PCA) performed on populations of *Tylenchulus semipenetrans* collected from citrus orchards in Fars province, focusing on the morphometric characteristics of the second-stage juveniles (A) and males (B).

For light microscopy, the extracted J2 of *T. semipenetrans* from the rhizosphere of citrus trees were killed and fixed in hot formaldehyde-acetic acid (4:1), and processed to anhydrous glycerol using Seinhorst’s method (1962). The morphological examination was carried out with an Olympus BX41TF microscope equipped with a camera at up to 1,000x magnification. The nematode population in the infected root was visualized and examined by staining the roots in fuchsin acid and subsequent clarification in acidified glycerol (Eisenback & Hunt, 2021). The morphometric indices of the males and J2s were also measured. The citrus nematode species was verified using *Tylenchulus* identification keys (Inserra et al., 1988; Tanha Maafi et al., 2012). To find out whether the populations have different morphometrics, a Principal Component Analysis (PCA) of the morphometric characters of males and J2 females of the nematode was performed in R version 3.5.1 (Fig. 2).

### DNA extraction

To extract genomic DNA, a single J2 of *T. semipenetrans* was hand-picked from the DESS solution (Yoder et al., 2006) using a pricking needle under a stereomicroscope and then washed thoroughly in distilled water. The nematode was placed in a 1.5-mL Eppendorf centrifuge tube containing 20 μL proteinase K (600 μg/ml) and 180 μL tissue lysis buffer (ATL) (two replicates for each population) and incubated overnight at 56°C. The extraction process was then continued according to the manufacturer’s instructions for the DNeasy Blood and Tissue Kit (QIAGEN, Hilden, Germany). The extracted DNA was either used directly or stored at −20° C in the DNA database of the Nematology Laboratory of the Czech University of Life Sciences, Prague.

### Primer selection and polymerase chain reaction (PCR)

For the analysis of the genetic diversity of *T. semipenetrans*, the DNA Sanger sequencing technique was performed. For this purpose, ITS and the D2-D3 segments of the 28S rDNA and COI mtDNA genes of 55 populations of the citrus nematode isolated from different districts were amplified (partial/complete), purified, and sequenced. For amplification of ITS rDNA (including ITS1, 5.8S and ITS2 regions), a universal primer pair of forward 18S and reverse 21S, as described by Marek et al. (2010), and a species-specific primer pair of forward Ts2-IF (TTCGAGAAACTTGGGGATTGGC) and reverse Ts2-IR (CAGGGACCTATGATCAAGTGCT) [presented in our study] were used. Since the amplification of the ITS rDNA of *T. semipenetrans* with the 18S and 21S primer pair was inefficient (Supplementary Fig. 1. A), this primer set was used as a basis for the development of a new primer pair (Ts2-IF: TTCGAGAAACTTGGGGATTGGC and Ts2-IR: CAGGGACCTATGATCAAGTGCT) with a smaller amplification product [770 bps] (Supplementary Fig. 1. B). The newly designed primer pair was developed based on the alignment of the sequences in the Vector NTI software (ThermoFisher Scientific) and the NCBI Primer-BLAST Tool (National Library of Medicine, Bethesda, MD). In addition, the designed primer pair fulfilled almost all criteria on the PCR Primer Stats website to be considered a suitable primer set (e.g., high GC content, low self-compatibility rate). To test the new primer set, the ITS rDNA sequences of common plant parasitic nematode species occurring in citrus orchards were obtained from NCBI and aligned in Mega 7 (https://www.kent.ac.uk/software/mega-7). The sequences were then searched for annotated primers. The forward primer (Ts2-IF) matched completely with two isolates of the citrus nematode, but at least five mismatches were detected for other taxa (including *Hemicycliophora* sp., *Mesocriconema* sp., *Paratylenchus* sp., *Gracilacus* sp., *Tylenchorhynchus* sp., *Hoplolaimus* sp., and *Meloidogyne* sp.) (Supplementary Fig. 2A). The result of the reverse primer search (Ts2-IF) in the ITS sequences of the other species showed that there were at least nine mismatches between the reverse primer sequence and the target sequences. Therefore, the ITS region of the above-mentioned taxa was not amplified with this primer set (Supplementary Fig. 2B). The sequences of the primer sets used in this study are listed in Table 2.

**Table 2:** The primers used in this study for identification and genetic diversity of *Tylenchulus semipenetrans* populations.

**Primer code**	**Primer sequence (5′-3′)**	**Product size (bp)**	**Target region**	**References**
18S	TTGATTAGGTCCCTGCCCTTT	967	ITS1-5.8S-ITS2	Marek et al., 2010
21S	TTTCACTCGCCGTTACTAAGG			
TW81F	GTTTCCGTAGGTGAACCTGC	809–841	ITS1-5.8S-ITS2	Tanha Maafi et al., 2003
AB28R	ATATGCTTAAGTTCAGCGGGT			
Ts2-IF	TTCGAGAAACTTGGGGATTGGC	770	ITS1-5.8S-ITS2	Present study
Ts2-IR	CAGGGACCTATGATCAAGTGCT		*T. semipenetrans* specific	
D2AF	ACAAGTACCGTGAGGGAAAGTTG	774–777	28S D2-D3	Subbotin et al., 2006
D3BR	TCGGAAGGAACCAGCTACTA			
COI-F5	AATWTWGGTGTTGGAACTTCTTGAAC	790	Cytochrome oxidase subunit I	Powers et al., 2014
COI-R9	CTTAAAACATAATGRAAATGWGCWACW			
	ACATAATAAGTATC-			

The total volumes of all PCR reactions were 20 μL, and contained 1 μL of DNA template; 0.4 μL forward and reverse primers mix 50 pmol μL^−1^ (Table 2); 10 μL Phusion HSII High Fidelity PCR Master Mix (17 μL ddH_2_O; 2.5 μL 10× buffer; 1.5 mM MgCl_2_); 1 U Taq DNA polymerase (200 μM each dNTP); and sterile ddH_2_O, added to a final volume of 20 μL. A negative control (without a DNA template) was also included in all reactions. Amplification was performed using a C1000 Touch Thermal Cycler (Biorad, Hercules, CA, USA). The thermocycling profile for the ITS rDNA gene consisted of an initial hot-start denaturation at 98°C for 30 s; 35 cycles of denaturation at 98°C for 10 s; 30 s of annealing [at 58°C with the 18S/21S primer pair and 56.5°C, using Ts2-IF/Ts2-IR primer pair]; and 72°C extensions for 35 s, followed by 72°C for 8 min to complete the process. The forward primer COIF5 and the reverse primer COIR9 (Powers et al., 2014) were used for amplification of the COI mtDNA gene (Table 2). PCR conditions for the COI gene consisted of 5 min at 94°C, followed by 40 cycles of 30 s at 94°C, 30 s at 48°C, and 90 s at 72°C, with a final extension at 72°C for 5 min. D2–D3 expansion of 28S rDNA was amplified using D2A and D3B (Subbotin et al., 2006) as forward and reverse primers, respectively (Table 2); the PCR program for this fragment included an initial treatment at 98°C for 30 s, 35 cycles at 98°C for 10 s, 58.4°C for 30 s, and a 72°C elongation step for 35 s, followed by an extension at 72°C for 8 min.

### DNA purification and sequencing

PCR products were run on a 1% TAE-buffered agarose gel (stained with ethidium bromide), visualized, and photographed under a UV transilluminator (80 V, 50 min). The high-volume PCR products were amplified (in 40 μL), run on a 1.5% TAE-buffered agarose gel, cut from the gel, and then purified using the GeneJET Gel Extraction kit (Fisher Scientific Lithuania, Vilnius) according to the manufacturer’s instructions. The proper fragments were sent to Eurofins Genomics (Ebersberg bei München, Germany) for sequencing in both directions with the corresponding primers. All newly obtained sequences were submitted to the GenBank database under the accession numbers listed in Table 1.

### Phylogenetic analysis

Before the alignment, the new DNA sequences obtained in this study were manually trimmed (if necessary) using Mega 7.1.0, and contiguous strands (contigs) were obtained using the online CAP3 sequence assembly program. A simple local alignment search tool in NCBI was used to check the species identity of the DNA sequences. Three datasets were created (one for each of the three sequenced genes) that contained the sequences of all collected populations, as well as closely related taxa. Taxa were selected based on the literature (Holterman et al., 2006; Subbotin et al., 2005, 2006; Bert et al., 2008; Rashidifard et al., 2015b). The available sequences and outgroups were aligned using MUSCLE (Edgar, 2004) implemented in Geneious Prime 2021.2.2 (www.geneious.com). According to jModeTest 2.1.10, General Time Reversible with a gamma distribution (GTR + G) was the best-fitting nucleotide substitution model for the ITS dataset and GTR was the best model for the D2-D3 and COI datasets (Darriba et al., 2012).

Bayesian analysis was performed using MrBayes version 3.1.2 (Ronquist & Huelsenbeck, 2003), which was used in Geneious Prime. For each of the datasets, the chain runs for 3 ×10^6^, after discarding 25% as burn-in samples. The Markov chain Monte Carlo (MCMC) method was used to estimate the posterior probabilities (PB) of the phylogenetic trees (Larget & Simon, 1999) using the 50% majority rule. A principal component analysis was performed in R version 3.5.1. to assess the dissimilarity between the sequences and the population measurements.

### Haplotype analysis

For haplotype analysis, the obtained consensus sequences were aligned with sequences from GenBank (if present) and screened for the presence of single nucleotide variations (SNV) and/or single nucleotide polymorphism (SNPs) using DnaSP Version 5.10.01 (Rozas et al., 2010). Further, nucleotide and haplotype diversity and other characteristics of the groups were evaluated. Moreover, the TCS haplotype network analysis and haplotype genealogy graphs, constructed using COI mtDNA genes and ITS sequences, were generated utilizing Hapsolutely version 0.2.2 (Clement et al., 2002; Vences et al., 2024).

## Results

### Morphometric assessment

The comparative morphometric data of males and J2 females of 30 populations of *T. semipenetrans* are shown in Tables 4 and 5. The characteristics were generally consistent with those reported for a population of *T. semipenetrans* from Florida (Inserra et al., 1988) and the previously reported population from Fars province (Rashidifard et al., 2015b). However, the populations in the present study differ from the Florida population and the other population from Fars province by the following morphometric indices means, respectively: slightly shorter male stylet (8.41 vs. 9.3 and 9 μm); shorter J2 stylet (11.4 vs. 12.3 and 13 μm); shorter male body length (347 vs. 362 and 368 μm); and shorter male tail (36 vs. 39.9 and 41.4 μm). Moreover, the body length of J2 was shorter than in the Florida population (332 vs. 363 μm) and slightly longer than in the Fars population (332 vs. 313 μm). Morphological identification of populations from Fars province in the present study was also verified by species-specific PCR using ribosomal DNA from J2. Moreover, the results of morphometric analysis of the characters based on Principal Component Analysis (PCA) showed that some populations have slightly different morphometry – for example, males and J2 females of populations 772 (Darab, orange trees) and 720 (Jahrom, sweet lemon) were located marginally apart from other populations. Also, males of 789 and 746 isolates and J2 of 777 isolates stood far from the other isolates (Fig. 2).

**Table 3:** Summary information of single nucleotide variations (SNV) and/or single nucleotide polymorphism (SNPs) analysis in this study for identification and genetic diversity of *Tylenchulus semipenetrans* populations from Fars province, Iran.

**Locus**	**No. of sites**	**Segregating sites or SNVs**	**SNPs**	**No. of haplotypes**	**Nucleotide diversity**	**Tajima’s D**	**Sequence conservation**	**Min recombination**
COI	603	16	11	10	Pi: 0.00767	D: 0.89661	C: 0.973	1
D2D3	676	24	12	33	Pi: 0.00535	D: −1.02740	C: 0.964	6
ITS	597	17	11	16	Pi: 0.00515	D: −0.89706	C: 0.972	2

**Table 4:** Morphometrics of males of 30 populations (five specimens each) of *Tylenchulus semipenetrans*, collected from citrus orchards of Fars Province, Iran. Data are given as mean ± standard deviation (range) of population means or specimens. Measurements are in μm.

**Characters**	**Means of populations**	**Specimens**
n	30	143
L	346 ± 13.2 (318.3–375)	347 ± 21 (296–424)
a	34.9 ± 1.9 (31.7–39.7)	34.9 ± 2.8 (29.6–44.9)
b	3.4 ± 0.2 (3.1–3.8)	3.5 ± 0.2 (2.8–4.3)
c	9.7 ± 0.5 (8.5–10.8)	9.7 ± 0.7 (8.0–12.1)
c’	4.7 ± 0.3 (4.1–5.5)	4.7 ± 0.5 (3.5–6.1)
Stylet	8.4 ± 0.4 (7.7–9.2)	8.4 ± 0.6 (7–11.1)^a^
Conus	4.9 ± 0.3 (4.3–5.7)	4.9 ± 0.5 (3.5–6.4)
Anterior end to the center of the median bulb	43.7 ± 3.2 (34.2–50)	43.7 ± 4.6 (31–64)
Pharynx length	100 ± 4.8 (90–111)	101 ± 7.6 (83–123)
Anterior end to hemizonid	65.6 ± 2.8 (58.6–70.6)	65.6 ± 4.1 (52–77)
Anterior end to nerve ring	61.1 ± 3.0 (53.0–66.7)	61.1 ± 4.2 (48.5–72.5)
Secretory-excretory pore (S. E. pore) to cloaca	100 ± 6.1 (87–110)	101 ± 8.9 (75–119.5)
Anterior end to S. E. pore	231 ± 20.7 (198–279)	229 ± 44 (161–333)
S. E. pore from the anterior end (% of L)	66.9 ± 5.3 (58.0–78.)	66 ± 12 (54–91.2)
Median bulb width	4.5 ± 0.6 (3.2–5.6)	4.5 ± 0.7 (2.7–6.2)
Median bulb length	11.2 ± 0.9 (9.3–13.0)	11.2 ± 1.4 (7.1–14)
Median bulb length/diam. Ratio	252 ± 30.3 (199–328)	251 ± 41 (176–385)
Max. body width (BW)	10.0 ± 0.5 (9.1–11.0)	10.0 ± 0.8 (8.3–13)
BW in Pharynx	8.8 ± 0.4 (8.0–9.6)	8.8 ± 0.6 (7.3–10.7)
Anal BW	7.7 ± 0.5 (7.3–10.2)	7.7 ± 0.9 (6.7–16.4)
Spicules (curved median line)	16.6 ± 0.7 (15.5–17.8)	16.6 ± 1.1 (14–20)
Gubernaculum	3.7 ± 0.3 (3.1–4.3)	3.7 ± 0.5 (2.5–5.0)
Tail	36.0 ± 2.4 (33.1–42.0)	36 ± 3.4 (29–47.5)
M	0.6 ± 0.0 (0.5–0.6)	60 ± 6 (41–77)
Stylet / L (%)	2.4 ± 0.1 (2.2–2.6)	2.4 ± 0.2 (2.1–3.2)
S. E. pore / L (%)	66.9 ± 5.3 (58.0–78.7)	66 ± 12 (53.9–91.2)

a: Stylet was greater than 10 μm in only four specimens

**Table 5:** Morphometrics of the second-stage juveniles of 31 populations (five specimens each) of *Tylenchulus semipenetrans*, collected from citrus orchards of Fars Province, Iran. Data are expressed as mean ± standard deviation (range) of population means or specimens. Measurements are in μm.

**Character/Population code**	**Means of populations**	**Specimens**
n	31	154
L	331 ± 12.1 (304–349)	332 ± 18 (276–376)
a	27.6 ± 1.1 (24.3–29.7)	28 ± 2 (22.4–34.7)
b	3.5 ± 0.1 (3.2–3.7)	3.5 ± .2 (3.1–4.1)
Stylet	11.4 ± 0.3 (11–11.8)	11.4 ± .4 (10.4–12.4)
Conus	6.1 ± 0.2 (5.5–6.7)	6.1 ± .4 (4.8–7.3)
Anterior end to the center of the median bulb	46.7 ± 1.6 (43.4–49.5)	46.8 ± 2.4 (39.9–53)
Pharynx length	94 ± 3.7 (85–103)	95 ± 5 (76–106.5)
MB	49.3 ± 1.2 (45.5–51)	49 ± 2 (41–55)
Anterior end to hemizonid	66.1 ± 2.2 (61.3–70.4)	66 ± 3.4 (58–77)
Anterior end to secretory-excretory pore (S. E. pore)	183 ± 7.5 (168–196)	183 ± 12 (147–222)
Anterior end to nerve ring	61.1 ± 2.3 (57.1–66.6)	61 ± 3 (51–71)
S. E. pore to genital primordium (GP)	19.4 ± 2.8 (12.8–24.9)	20 ± 4.2 (12–32)
Anterior end to GP	200 ± 6.7 (184–212)	200 ± 10 (165–222)
GP length	12.6 ± 1.6 (9.8–17.4)	13 ± 2 (8.5–21)
GP to the posterior end	118 ± 7.4 (101–130)	119 ± 11.7 (90–155)
Excretory pore from the anterior end (% of L)	56.2 ± 2.1 (53.2–60.4)	56 ± 3 (47–64)
Anterior end to GB (% of L)	60.6 ± 1.3 (58.4–63.2)	60 ± 2 (54–67)
Median bulb width	6.2 ± 0.3 (5.4–6.6)	6 ± .5 (4.9–7.5)
Median bulb length	13.2 ± 0.9 (11.9–16)	13 ± 1.4 (10–18)
Median bulb length/diam. ratio	211 ± 16.5 (182–262)	212 ± 26 (104–282)
body width (BW) in Pharynx	11.2 ± 0.4 (10.3–11.9)	11 ± .6 (9.6–13)
Max. BW	12.0 ± 0.4 (10.7–12.6)	12 ± .6 (10–14)
M	0.5 ± 0.02 (0.5–0.6)	.5 ± 0 (.4–.7)
Stylet / L (%)	3.5 ± 0.1 (3.2–3.9)	3.4 ± .2 (3–4)
S. E. pore / L (%)	55.4 ± 2.1 (51.8–60)	55 ± 3 (47–64)

### Phylogenetic and haplotype analyses using ITS, D2-D3 28S and COI genes

In the present study, a total of 134 new sequences of *T. semipenetrans* were obtained, including 48 sequences each of ITS rDNA, D2-D3 of 28S rDNA, and COI mtDNA (Table 1). Based on BLAST search, the sequences of ITS and D2-D3 rDNA matched the sequences of *T. semipenetrans* available in GenBank, with similarities ranging from 96.3% to 100%. There was no deposited sequence of COI mtDNA for the citrus nematode in the database, so the sequences were aligned with other taxa in the superfamily Criconematoidea – e.g., *Hemicriconemoides macrodorus* (KM577167), *Hemicriconemoides promissus* (KM577164), *Ogma seymouri* (MN711327 & MN711328), and *Crossonema menzeli* (MN710911 & MN710914), with a similarity level higher than 86%. The results indicate a low variability among the analyzed isolates.

### ITS rDNA

The electrophoretic separation of the amplified ITS rDNA resulted in a single product size of 770 bp for all samples. After aligning the sequences of the ITS dataset, 597 bp were used for phylogenetic analysis. Alignment of the ITS consensus sequences of our *T. semipenetrans* populations with a reference sequence of *T. semipenetrans* from GenBank (JN112270) revealed the presence of 17 single nucleotide variations (SNVs), 11 of which were identified as SNPs (Table 3). The results showed that on average one nucleotide variation occurred per 31 base pairs. Additional information about the DnaSP analysis and the location of the mutations are shown in Tables 3 and 6. The most frequent mutation was the transition from cytosine to thymine (with seven transitions located at nucleotides 92, 151, 161, 225, 405, 423 and 523), followed by the transition from guanine to adenine (three transitions situated at nucleotides 37, 46 and 139) (Table 6).

**Table 6:** Single nucleotide polymorphism in the alignment of the citrus nematode (*Tylenchulus semipenetrans*) ITS of rDNA gene partial sequences.

**ITS haplotypes**	**Isolate(s)**	**Position of the single nucleotide variations/polymorphisms (SNVs/SNPs) on the sequences**																
		**32**	**37**	**46**	**71**	**92**	**122**	**139**	**151**	**159**	**161**	**162**	**188**	**225**	**354**	**405**	**423**	**523**
***T. semipenetrans* JN112270.1**	**CD1_cl2**	**A**	**G**	**G**	**T**	**C**	**A**	**G**	**C**	**A**	**C**	**T**	**T**	**C**	**T**	**C**	**C**	**C**

TsA (n = 20)	37, 42, 112, 678, 706, 716, 717, 733, 743, 755, 763, 777, 778, 789, 795, 821,882, 921 BEHZ & SH2	A	G	G	T	C	A	G	C	A	C	T	T	C	T	C	C	C
TsB (n = 6)	707, 710, 754, 801, 818 & ARE	C	A	G	A	C	A	G	C	A	C	T	T	C	T	C	C	C
TsC (n = 1)	812	C	A	G	T	C	A	G	C	A	C	C	T	C	T	C	C	C
TsD (n = 3)	720, 773 & 802	C	A	A	A	C	A	C	C	A	C	T	T	C	T	C	C	C
TsE (n = 1)	785	C	A	G	A	C	A	A	C	A	C	T	C	T	T	C	C	C
TsF (n = 1)	759	C	A	G	A	T	A	A	C	A	C	T	C	C	T	C	C	C
TsG (n = 6)	712, 735, 737, 749, 771, & 908	A	G	G	T	C	A	G	C	A	C	C	T	C	T	C	C	C
TsH (n = 1)	25	C	A	G	A	T	A	A	C	A	T	T	C	C	T	C	C	C
TsI (n = 1)	BEH	A	G	G	T	C	A	G	C	A	C	T	T	T	T	C	C	C
TsJ (n = 1)	AMI	A	G	G	T	C	A	G	C	A	C	T	C	C	T	C	C	C
TsK (n = 1)	746	C	A	G	A	T	A	A	C	A	C	T	C	C	A	T	C	T
TsL (n = 1)	793	C	A	G	A	C	A	G	C	G	C	T	T	C	T	C	C	C
TsM (n = 1)	411	C	A	G	T	T	A	A	C	A	C	T	C	C	T	C	C	C
TsN (n = 1)	772	C	A	G	T	C	A	G	T	T	C	T	T	C	T	C	T	T
TsO (n = 1)	32	C	A	G	A	T	G	A	C	A	C	T	C	C	T	C	C	C
TsP (n = 1)	780	C	G	G	T	C	A	G	C	A	C	C	T	C	T	C	C	C

**Figure 3:**
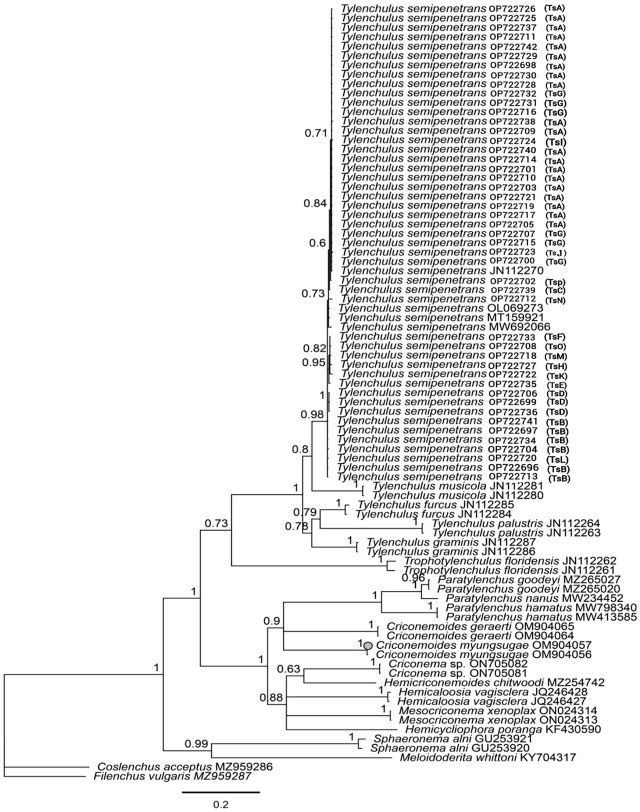
Bayesian phylogenetic tree of *Tylenchulus semipenetrans* isolates from citrus orchards in Fars province based on the ITS of rDNA sequences, analyzed under the General Time Reversible with a gamma distribution (GTR + G) model. Numbers at nodes are posterior probability values. Sequences with codes in the parentheses generated in this study. The codes in parentheses indicate the haplotype of the relevant population.

The phylogenetic relationships of *T. semipenetrans* populations based on the ITS rRNA gene are shown in Figure 3. Isolates of five known *Tylenchulus* species were well-delineated in the tree and formed five distinct subclades. The main clade, which included all species of *Tylenchulus*, was closely related to another clade that included two isolates of *Trophotylenchulus floridensis* (JN112261 and JN112262) (PB: 0.73). All newly obtained sequences of *T. semipenetrans* grouped with those of the same species from GenBank in a strongly supported subclade (BP: 0.97), and *T. musicola* was the closest species to *T. semipenetrans*.

### D2-D3 fragments of the 28S rDNA

PCR of the D2-D3 fragments of the 28S rDNA yielded amplification products 774–777 bp in length which, was obtained from a single J2 of *T. semipenetrans*. Comparison of 48 sequences of this gene in the present study with a reference sequence of *T. semipenetrans* from GenBank (KM598334.1) resulted in an alignment 676 bp in length. The alignment showed 24 segregating sites, or SNVs, of which 12 were SNPs (Table 7). In other words, there was an average of one segregating site per 28 bp of the D2D3 28S sequences (Table 3). The most common SNV was cytosine-to-thymine transition (seven, at positions 55, 84, 157, 260, 263, 355, and 497), followed by thymine-to-cytosine transition (four, at positions 59, 94, 238, and 502) and guanine-to-thymine transversion (four, at positions 34, 119, 340 and 369).

**Table 7:** Single nucleotide polymorphism in the alignment of the citrus nematode (*Tylenchulus semipenetrans*) D2-D3 of 28 S rDNA gene.

**D2D3 haplotypes**	**Isolate(s)**	**Position of the single nucleotide variations/polymorphisms (SNVs/SNPs) on the sequences**																							
		**34**	**55**	**59**	**73**	**84**	**94**	**104**	**113**	**119**	**125**	**157**	**167**	**223**	**238**	**260**	**263**	**340**	**355**	**369**	**456**	**497**	**502**	**616**	**661**
***T. semipenetrans* KM598334.1**	**ES-Jirof2**	**G**	**C**	**T**	**C**	**C**	**T**	**A**	**G**	**G**	**A**	**C**	**G**	**T**	**T**	**C**	**C**	**G**	**C**	**G**	**A**	**C**	**T**	**G**	**A**

Ts1 (n = 6)	720, 773, 32, 759, 765 & 818	G	C	T	C	C	T	A	G	G	A	C	G	T	T	C	C	G	C	G	A	C	T	G	A
Ts2 (n = 2)	BEH & 921	G	C	T	C	T	T	A	G	G	A	C	C	T	T	C	C	T	C	G	A	T	T	G	A
Ts3 (n = 2)	AMI & 812	G	C	T	C	T	T	A	G	G	A	C	C	T	T	C	C	T	C	G	A	C	T	G	A
Ts4 (n = 1)	755	G	C	T	C	C	T	A	G	G	A	C	G	G	C	C	C	G	C	G	G	C	T	G	A
Ts5 (n = 1)	882	G	C	T	C	C	T	A	G	G	A	C	G	T	C	C	C	G	C	G	G	C	T	A	A
Ts6 (n = 1)	802	G	C	T	C	T	T	A	G	G	A	C	G	T	T	C	C	T	C	T	A	C	T	G	A
Ts7 (n = 1)	785	G	C	T	C	C	T	A	A	G	A	C	G	T	T	C	C	G	C	G	A	C	T	G	A
Ts8 (n = 1)	ARE	G	C	T	C	T	T	A	G	G	A	C	C	T	T	C	C	T	T	G	A	T	T	G	A
Ts9 (n = 2)	740 & 749	G	C	T	C	C	T	A	G	G	G	C	G	T	T	C	C	G	C	G	A	C	T	G	A
Ts10 (n = 1)	712	G	C	T	C	C	T	A	G	G	G	T	G	T	T	C	C	G	C	G	A	C	T	G	A
Ts11 (n = 1)	678	G	C	T	C	T	T	A	G	G	A	C	G	T	C	C	C	G	C	G	A	C	T	G	A
Ts12 (n = 2)	112 & 777	G	C	T	C	T	T	A	G	G	A	C	C	T	T	C	T	T	C	G	A	T	T	G	A
Ts13 (n = 1)	746	G	C	T	C	C	T	T	G	G	G	T	G	T	T	C	C	G	C	G	A	C	T	G	A
Ts14 (n = 1)	42	G	C	T	C	T	T	A	G	G	A	C	G	T	T	C	C	T	C	G	A	C	T	G	A
Ts15 (n = 1)	25	G	C	T	C	C	T	A	G	T	G	C	G	T	T	C	C	G	C	G	A	C	T	G	A
Ts16 (n = 9)	717, 698, 706, 716, 789, 821, 743, 763 & SH2	G	C	T	C	C	T	A	G	G	A	C	G	T	C	C	C	G	C	G	G	C	T	G	A
Ts17 (n = 2)	733 & SH1	G	C	T	C	C	T	A	G	G	A	C	G	T	C	C		G	C	G	A	C	T	G	A
Ts18 (n = 1)	BEHZ	G	C	T	C	C	T	A	G	G	A	C	C	T	T	C	C	T	C	G	A	C	T	G	A
Ts19 (n = 1)	795	G	C	T	C	T	T	A	G	G	A	C	G	T	C	C	C	T	C	G	A	C	T	G	A
Ts20 (n = 2)	754 & 793	G	C	T	C	C	T	A	G	G	G	T	G	T	T	T	C	G	C	G	A	C	T	G	A
Ts21 (n = 1)	411	G	C	T	C	C	T	A	G	G	A	C	G	T	T	C	C	G	C	G	A	C	C	G	A
Ts22 (n = 1)	737	G	C	T	C	T	T	A	G	G	A	C	G	T	T	C	C	G	C	G	A	C	T	G	A
Ts23 (n = 1)	735	G	C	T	C	T	T	A	G	G	G	T	G	T	T	C	C	T	C	G	A	C	T	G	A
Ts24 (n = 1)	801	G	C	T	C	T	T	A	G	G	A	C	C	T	T	C	C	T	C	T	A	T	T	G	A
Ts25 (n = 1)	707	G	C	T	C	T	T	A	G	G	A	C	G	T	C	C	C	G	C	T	A	C	T	G	A
Ts26 (n = 1)	772	T	C	C	G	T	T	A	G	G	A	C	G	T	T	C	C	T	C	G	A	C	T	G	A
Ts27 (n = 1)	710	G	C	T	C	T	T	A	G	G	A	C	C	T	T	C	C	G	C	G	A	C	T	G	A
Ts28 (n = 1)	37	G	T	C	C	C	T	A	G	G	A	C	G	T	T	C	C	G	C	G	A	C	T	G	A
Ts29 (n = 1)	908	G	C	T	C	C	C	A	G	G	G	C	G	T	T	C	C	G	C	G	A	C	T	G	A
Ts30 (n = 1)	788	G	C	T	C	T	T	A	G	G	A	C	G	T	C	C	C	G	C	G	G	C	T	G	A
Ts31 (n = 1)	780	G	C	T	C	C	T	A	G	G	G	T	G	T	T	C	C	G	C	T	A	C	T	G	A
Ts32 (n = 1)	778	G	C	T	C	T	T	A	G	G	A	C	C	T	C	C	C	G	C	G	A	C	T	G	A
Ts33 (n = 1)	771	G	C	T	C	C	T	A	G	G	G	T	G	T	T	C	C	G	C	G	A	C	T	G	C

**Figure 4:**
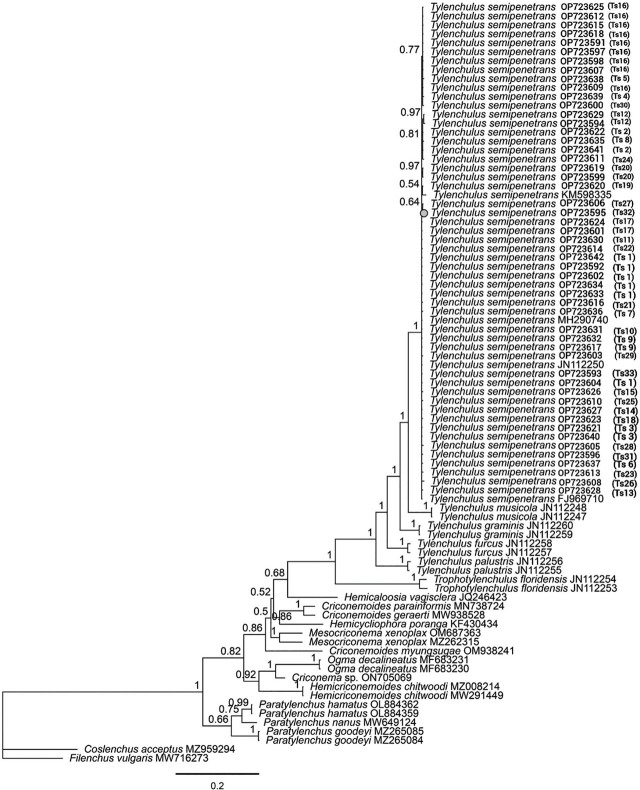
Bayesian phylogenetic tree of *Tylenchulus semipenetrans* isolates from citrus orchards in Fars province based on D2-D3 28S rDNA partial sequences, analyzed by the General Time Reversible (GTR) model. The numbers shown at the nodes are posterior probability values. Sequences with codes in the parentheses generated in this study. The codes in parentheses indicate the haplotype of the relevant population.

Phylogenetic studies for the citrus nematode isolates based on D2-D3 rDNA sequences are shown in Figure 4. Similar to the ITS tree, all isolates of *T. semipenetrans* formed a separate, maximally supported subclade (BP: 1.00) from the other known species of the genus. *Tylenchulus musicola* was the closest species to *T. semipenetrans* based on the D2-D3 sequences. The main clade, which included all species of *Tylenchulus*, was closely related to another clade comprising *Trophotylenchulus floridensis* isolates (JN112253 and JN112254).

### Cytochrome oxidase subunit 1

This study provided the first sequences of the cytochrome oxidase subunit 1 (COI) gene for *T. semipenetrans* from Iran. The PCR reaction for the COI mtDNA gene of the citrus nematode populations yielded a single fragment of 790-bp nucleotides. The 603-bp alignment of 48 COI mtDNA gene sequences of our *T. semipenetrans* isolates revealed 16 single nucleotide variations, corresponding to an average of one variation per 38 bp. Out of 16 segregating sites (or SNVs), 11 were identified as SNPs (Tables 3 and 8). The most common mutation was the transition of adenine to guanine, with five transitions at nucleotides 406, 481, 515, 535 and 574.

**Table 8:** Single nucleotide polymorphism (SNPs) in the alignment of the citrus nematode (*Tylenchulus semipenetrans*) COI mtDNA partial gene.

**COI haplotypes**	**Isolate(s)**	**Position of the single nucleotide variations/polymorphisms (SNVs/SNPs) on the sequences**															
		**49**	**250**	**353**	**385**	**393**	**406**	**425**	**448**	**469**	**481**	**515**	**532**	**535**	**562**	**574**	**580**
TsI (n = 21)	771, 908, 32, 710, 801, 735, 737, 793, 795, 746, AMI, BEHZ, 25, 42, 112, 749, 759, ARE, 802, 25 & 812	C	G	A	T	A	A	T	A	T	A	A	G	A	C	A	T
TsII (n = 10)	698, 754, 707, 733, 716, 763, BEH, 712, 765 & 921	T	A	A	C	A	A	T	A	A	A	A	A	A	C	A	T
TsIII (n = 11)	720, 772, 773, 821, 743, 411, SH1, SH2, 785, 755 & 818	T	A	A	T	A	A	T	T	A	G	A	A	G	T	G	T
TsIV (n = 1)	740-2	T	A	A	C	A	A	T	T	A	G	A	A	G	T	G	T
TsV (n = 1)	789	T	A	A	C	A	A	T	A	A	A	A	A	A	T	G	T
TsVI (n = 1)	706	C	G	A	T	A	A	T	A	T	A	G	G	A	C	A	T
TsVII (n = 1)	780	C	G	A	T	C	G	T	A	T	A	A	G	A	C	A	T
TsVIII (n = 1)	778	C	G	C	T	A	A	T	A	T	A	G	G	A	C	A	T
TsIX (n = 1)	777	C	G	A	T	A	A	T	A	T	A	A	G	A	C	A	G
TsX (n = 1)	717	C	G	A	T	A	A	G	A	T	A	A	G	A	C	A	T

**Figure 5:**
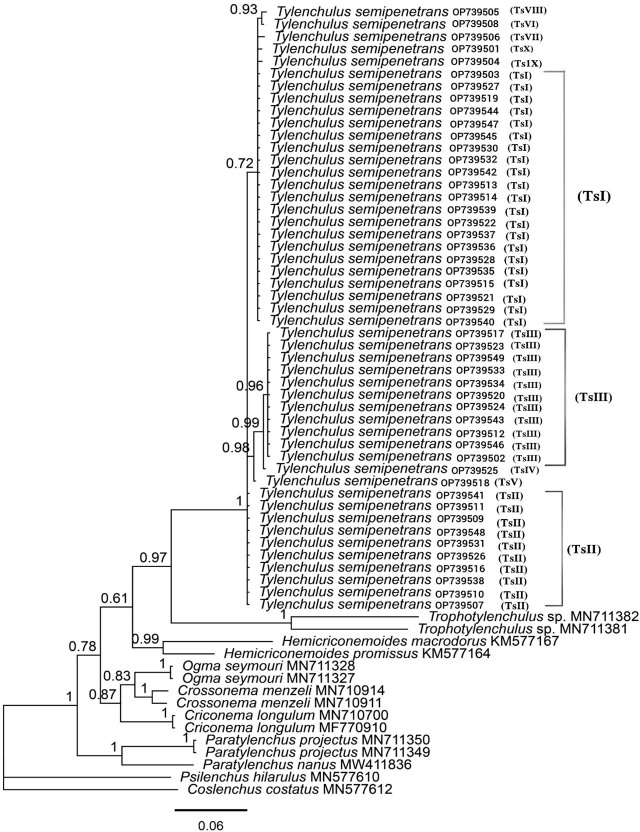
Bayesian phylogenetic tree of *Tylenchulus semipenetrans* isolates from citrus orchards of the Fars Province based on COI mtDNA partial sequences, analyzed under the General Time Reversible (GTR) Model. Numbers shown on nodes are posterior probability values. All *T. semipenetrans* sequences were produced in this study. The codes in parentheses indicate the haplotype of the relevant population.

The phylogenetic relationships of *T. semipenetrans* populations based on the COI mtDNA gene revealed that all isolates of *T. semipenetrans* formed a strongly supported clade (BP: 1.00); this clade was in a well-supported sister relationship with two unidentified species of *Trophotylenchulus* (MN711381 and MN711382) (BP: 0.97). No COI sequence was available for other known species of *Tylenchulus* (Fig. 5).

### Haplotypes

**Figure 6:**
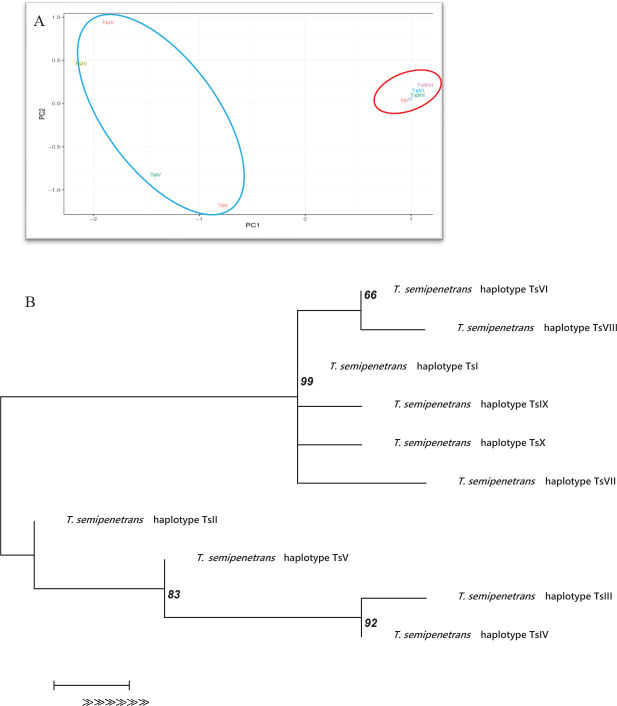
A: principal component analysis (PCA) generalized linear modeling of *Tylenchulus semipenetrans* haplotypes from citrus orchards of the Fars province based on COI mtDNA, and B: the corresponding phylogenetic tree, analyzed under the Hasegawa Kishino Yano (HKY) model in MEGA 7. Numbers shown on nodes are posterior probability values.

Based on the occurrence of SNV among the sequences of *T. semipenetrans*, haplotypes corresponding to each of the genes are presented in Tables 6–8. Preliminary principal component analysis of the SNV level of the haplotypes based on all genes was performed separately. PCA analysis revealed distinct groupings within the COI mtDNA gene (Fig. 6). In contrast, the ITS and D2D3 genes did not exhibit clear clustering patterns, so data for these genes are not presented. The haplotypes of *T. semipenetrans* based on the COI mtDNA gene were TsI, TsII, TsIII, TsIV, TsV, TsVI, TsVII, TsVIII, TsIX and TsX. The TsI, TsIII and TsII haplotypes were more frequent than others (Table 8 and Fig. 5). The concordance between the PCA biplot and the corresponding phylogenetic tree of haplotypes was observed (Fig. 6). This congruence supported the clustering of TsII, TsIII, TsIV, and TsV, while other haplotypes formed a distinct group (Fig. 6). The TCS network analysis also indicated that there was little variation among the samples (Supplementary Fig. 3).

## Discussion

In this study, we performed a phylogenetic analysis of *T. semipenetrans* within representatives of the genus and the family Tylenchulidae using 134 new sequences from three different genes including 28S and ITS rDNA, as well as COI mtDNA. There were few nucleotide differences within the populations of *T. semipenetrans* (Tables 6–8). The lack of nucleotide differences among the isolates tested indicated a low genetic diversity among *T. semipenetrans* populations worldwide and suggested that the citrus nematode is a genetically homogeneous species. This is consistent with the results reported by Tanha Maafi et al. (2012) and Rashidifard et al. (2015b), which also found a low degree of sequence dissimilarity in Iranian populations of *T. semipenetrans*. The results of this study provided the first partial sequence of the COI mtDNA gene, along with new sequences of the D2-D3 expansion and the ITS region of the citrus nematode, in Fars province.

**Figure 7:**
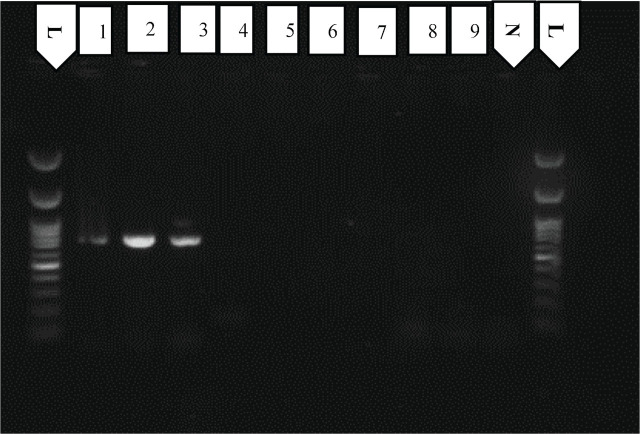
Agarose gel electrophoresis verification of amplified products of ITS rDNA reactions using forward Ts2-IF and reverse Ts2-IR. The lane labeled as follows: Negative control (N), *T. semipenetrans* (1–3), *Tylenchorhynchus* sp. (4 & 5), *Mesocriconema* sp. (6 & 7), *Hemicycliophora* sp. (8 & 9), and DNA ladder (L).

In addition, a new species-specific primer set (Ts2-IF and Ts2-IR) was designed in the present study to facilitate the amplification of 770 bp of the ITS rDNA of *T. semipenetrans*. The newly designed primers were based on the search for a new, larger (770 bp vs. 113 bp), and more conservative region of the ITS gene from all isolates of the citrus nematode. These primers could become a useful tool for the accurate and rapid identification of different isolates of *T. semipenetrans*. The newly designed primer set should be tested against other *Thylenculus* species in the world, but those other species are not cosmopolitan and are only related to the geographical area where they have reported for the first time, making them difficult to access. Consequently, the new primer set was tested against common plant-parasitic nematode species occurring in citrus orchards that are taxonomically related to the citrus nematode, such as *Hemicycliophora* sp., *Mesocriconema* sp. and *Tylenchorhynchus* sp. (Fig. 7).

According to analysis of the small ribosomal DNA (SSU rDNA) subunit, the superfamily Criconematoidea consists of the three families: Criconematidae, Hemicycliophoridae and Tylenchulidae (De Ley & Blaxter, 2002). The phylogeny of this group was evaluated by Subbotin et al. (2005) based on morphological and biological characters and the D2-D3 sequences of the 28s rDNA. Based on their results, the sedentary nematodes of this group, including *Trophonema*, *Sphaeronema* and *Tylenchulus*, were placed in a separate clade. In our phylogenetic trees, the isolates of *Tylenchulus* formed sister clades with representatives of *Trophotylenchulus*, indicating the close relationship of these two genera. Considering the available data sets, the validity of the five known species of *Tylenchulus* in the ITS and 28S trees was confirmed as they occupied close but different phylogenetic positions in their respective trees. In addition, our study has explicitly shown that *T. semipenetrans* is a monophyletic species, and that *T. musicola* is phylogenetically the closest species to it.

Based on the phylogenetic trees inferred from the different genes, there was no difference among the recovered populations of the citrus nematode. Differences appeared only after the intraspecific genetic variability was evaluated using DnaSP analysis, such that 17, 24 and 16 single nucleotide variations (SNVs) were observed in ITS, 28S, and COI genes, respectively. Most SNVs, detected in more than one of the investigated populations, were assessed as single nucleotide polymorphisms (SNPs) (Bilska-Zajac et al., 2019). Accordingly, 11, 12, and 11 SNPs were recognized in the ITS, 28S and mtDNA genes, respectively. Since genetic variations could be associated with biological or pathogenic traits, point mutations in a gene sequence and their translations to the corresponding amino acids can alter protein function and lead to unpredictable characteristics in organisms. In our data, however, transition mutations [interchanges of A ↔ G or C ↔ T] were detected at a significantly higher frequency than transversions (e.g., interchanges of purine for pyrimidine bases) (Tables 6–8). It seems that the impact of transition mutations on the alteration of the biological features of an organism is smaller than that of transversion mutations. Since transitions are less likely to result in amino acid substitutions, they are therefore more likely to persist as silent mutations (Zou & Zhang, 2021).

Preliminary haplotype analysis based on the SNVs revealed 16 (TsA-TsP), 33 (Ts1-Ts33), and 10 (TsI-TsX) haplotypes in the ITS and D2-D3 segments of 28S DNA and COI mtDNA, respectively (Tables 6–8). Some nucleotide sequences in GenBank are 100% identical to haplotypes in our study – e.g., the sequence found in orange from the USA (JN112270.1) was turned out to be the same as the TsA of *T. semipenetrans*. Also, nucleotide sequences of the isolates collected from northern Iran (SHI and SHII) were similar to those from Fars province in southern Iran (haplotypes Ts17 and Ts16, respectively). These findings suggest that the haplotype topology of the citrus nematode population does not correlate with geographic location.

Furthermore, there is no clear evidence of whether the citrus nematode is native or was introduced to Fars province. In many instances, populations collected from different localities are grouped in the same haplotype as specified by Dnasp analysis. For example, the TsA haplotype (based on D2D3) included isolates from various localities of Fars province in the south of Iran (Shiraz, Kazerun, Fasa, Khafr, Jahrom, Darab, Ghir, Karzin) and samples from Mazandaran province in the north. Moreover, this haplotype (TsA) was identical to a sequence of *T. semipenetrans* from GenBank (KM598334) (Tables 1 and 6–8). The findings imply that the citrus nematode may have spread further from an original introduction site via infected planting material or the recent flooding in Fars citrus orchards (Asrari & Masoudi, 2010). The data also demonstrated no clear correlation between genetic variability or haplotypes and host type; for instance, *T. semipenetrans* isolates from sour orange, orange, sweet lemon, and bitter orange were all assigned to the same haplotype (TsA) (Tables 1 and 6–8).

We employed PCA at the SNV level. The PCA analysis based on COI mtDNA revealed a close relationship between TsI, TsVI, TsVII, and TsVIII haplotypes. The PCA results also showed a weak correlation between the geographical origin of the host and specific haplotypes (Fig. 6).

In addition to using molecular data, morphological and morphometric differences are also considered important in distinguishing PPN populations and species. Therefore, the morphometrics of the J2 and males were also examined. Several *Tylenchulus* populations (Table 1) were identified as *T. semipenetrans* based on comparison of their morphological and morphometric characteristics (Tanha Maafi et al., 2012). The populations were morphologically very similar except for some indices, so they could not be separated. The stylet length of J2, a key index in distinguishing nematode species (Brzeski, 1991; Sturhan & Brzeski, 1991), was shorter, at 11.4 (10.4–12.4) μm, than in the data published by Inserra et al. (1988) [12.4 (12.2–13.2) μm] and Rashidifard et al. (2015b) [13 (11–17) μm]. The stylet length of males was also shorter, at 8.41 (7–11.1) μm, than that found in the data published by Inserra et al. (1988) (9.3 [9.1–10.2] μm) and Rashidifard et al. (2015b) (9 [7–10] μm), but was within the minimum and maximum ranges. Partial variation in morphometric characteristics among PPN populations could be due to their geographic distribution. However, the results showed that morphometric characters among and within populations of *T. semipenetrans* were not correlated with their geographic origin. Moreover, the morphometrics of the populations (J2 and male specimens) were generally within the ranges reported for these populations (Inserra et al., 1988). Therefore, we hypothesize that the slight morphometric differences observed among populations of *T. semipenetrans* may be variations or demonstrations of the phenotypic plasticity typical of nematodes (De Oliveira et al., 2017). PCA analysis based on the morphometric characteristics of males and J2 females showed slight differences among populations. Nevertheless, there was no obvious correlation between the morphometric and sampling areas of the populations (Fig. 2).

In conclusion, the high morphometric similarity of the 46 populations of *T. semipenetrans* collected from different localities in Fars province indicates the lack of variation, or the phenotypic plasticity, typical of nematodes. The phylogenetic studies of ITS rDNA, D2-D3 of 28S rDNA, and COI mtDNA gene sequences revealed no significant differences among the populations of *T. semipenetrans*. However, the comparison of the aligned consensus sequences of the genes (new sequences in this study and the reference sequence from GenBank) revealed the presence of single nucleotide variations or polymorphisms (SNVs/SNPs) and haplotypes. Sequence alignment showed 16, 33 and 10 haplotypes based on the ITS, D2-D3, and COI genes, respectively. A PCA biplot based on the COI mtDNA and the corresponding phylogenetic relationship of the haplotypes showed two separate groups. It seems that most of the variations are silent mutations, as transitions were found more frequently than transversions. The morphometric differences and haplotype topology observed among and within the populations were not related to their geographic and/or host background. Taking into account the accessibility of sequences in GenBank for comparison, *T. musicola* was the closest among the species most closely related to *T. semipenetrans*. The results of the present study may contribute to the development of citrus nematode control strategies. They can also serve as a basis for other researchers in the study of the genetic variability of this nematode.
